# Modification of Asphalt Rubber with Nanoclay towards Enhanced Storage Stability

**DOI:** 10.3390/ma11112093

**Published:** 2018-10-25

**Authors:** Jiangmiao Yu, Zhibin Ren, Huayang Yu, Duanyi Wang, Shekhovtsova Svetlana, Evgeniy Korolev, Zheming Gao, Feng Guo

**Affiliations:** 1 School of Civil Engineering and Transportation, South China University of Technology, Wushan Road, Tianhe District, Guangzhou 510000, China; yujm@scut.edu.cn (J.Y.); mszhibinren@mail.scut.edu.cn (Z.R.); tcdywang@scut.edu.cn (D.W.); 2 Institute of Construction and Architecture, Moscow State University of Civil Engineering, Yaroslavskoe Shosse, Moscow 129337, Russia; rusina.svetlan@yandex.ru (S.S.); KorolevEV@mgsu.ru (E.K.); 3 Sonny Astani Department of Civil and Environmental Engineering, University of Southern California, Los Angeles, CA 90098, USA; zhemingg@usc.edu; 4 Department of Civil Engineering and Environment, University of South Carolina, Columbia, SC 29205, USA; fengg@email.sc.edu

**Keywords:** storage stability, asphalt rubber, nanoclay, rheological properties

## Abstract

Asphalt rubber (AR), which is prepared by blending crumb rubber and bitumen, provides various advantages, including superior rutting resistance, lower road-tire noise and longer service life. However, contractors have expressed concerns regarding its poor storage stability, which in turn limits its wider application. This study aims to address the storage stability concern by incorporating nano-montmorillonite (nanoclay). Three types of nanoclay were dispersed into hot AR binder by high shear blending. The rheological properties of nanoclay-crumb rubber modifier (CRM)-modified bitumen were evaluated through Superpave performance grade (PG) tests and the storage stability was characterized by measuring the difference in softening points or complex moduli at the top and bottom portions of binders after lab-simulated storage. X-ray diffraction (XRD) evaluation was conducted to observe the variation of nanoclay layer gap distance for mechanism investigation. It was found that all selected nanoclays had insignificant effects on workability, rutting and fatigue properties. The layered nanoclay transformed to intercalated or exfoliated structures after interaction with bitumen fractions, providing superior storage stability. Among the three selected nanoclays, pure montmorillonite with Na^+^ inorganic group, which has an intermediate hydrophilic property and middle layer gap, showed the most obvious effect on enhancing the storage stability of AR.

## 1. Introduction

During the past few decades, the application of asphalt binder modifiers to enhance the overall performance of bitumen pavements has been a popular topic in pavement engineering [1,2,3,4,5,6]. From both the economic and environmental standpoints, crumb rubber modifier (CRM) from waste vehicle tires is considered as a beneficial and effective modifier [6,7,8,9]. Asphalt rubber (AR), which is prepared by blending crumb waste tire rubber and bitumen, has been proven to provide various advantages in comparison to conventional asphalt binder, such as superior rutting resistance, lower road-tire noise and longer service life [10]. However, poor workability and storage stability limit AR’s wider application. The workability concern is caused by the interaction among CRM and bitumen liquid phases, which can be effectively alleviated by using warm mix asphalt (WMA) technology [11,12]. The poor high-temperature storage stability of AR is caused by the density difference between neat bitumen and the incorporated CRM particles. However, few practical approaches have been proposed for the storage stability concern of AR.

The phase separation phenomenon, which causes poor storage stability, is governed by Stoke’s law [13,14,15], as shown below.

(1) $$v_{t}=\frac{2a^{2}\Delta \rho g}{9\eta }\;$$

where *v_t_* is the settling velocity of dispersed particles, α is the radius of dispersed particles, Δρ is the density difference between two different phases, *g* is gravitational acceleration, and *η* is the dynamic viscosity of liquid medium.

According to Equation (1), during the storage period (in elevated temperature), insoluble rubber particles in liquid AR binder tend to sink, which may eventually result in a thick congealed layer of rubber-rich phase at the bottom of CRM-bitumen blends. To address the storage stability concern of polymer-modified binders, one potential method is to reduce the settling velocity of dispersed rubber particles in the bitumen-rich phase by incorporating nanoclay materials, which may decrease the driving force of separation between polymer and neat bitumen by their unique physical properties [16,17].

**Figure 1:**
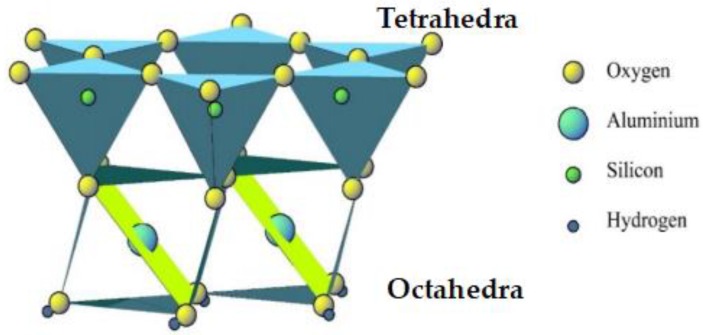
Structure of montmorillonite nanoclay [17].

Nanoclays are naturally occurring minerals that mainly include kaolinite clay (KC), vermiculite (VMT) and montmorillonite (MMT) [18]. Most commercial clay materials are alumino-silicates with layered structures, which consist of silica SiO_4_-tetrahedron bonded to alumina AlO_6_-octahedron with different patterns [19]. Figure 1 shows a typical internal structure of MMT nanoclay, which is composed of tetrahedral silicate layers and octahedral alumina layers. It has been reported that KC, VMT and MMT can be used as bitumen modifiers, as the polymer chains in asphalt binder may intercalate into the interlayer of clay, which makes the clay tightly bonded to the polymer matrix at nano scale, leading to significantly enhanced mechanical performance [20,21,22]. For example, the MMT-modified bitumen may form an intercalated structure, while organophilic montmorillonite (OMMT)-modified bitumen may form an exfoliated structure [22]. Furthermore, although the density of nanoclay is larger than asphalt binder, the intercalated or exfoliated structure between clay and neat bitumen may prevent the sedimentation of nano particles. As a result, nanoclays have also been used for improving the storage stability of polymer-modified bitumen.

The objective of this study is to enhance the storage stability of asphalt rubber by incorporating different nanoclays. To this end, the rheological properties of nanoclay-CRM-modified bitumen were evaluated by Superpave Performance Grading (PG) tests and the storage stability was characterized by measuring the difference between the top and bottom portions of binders after lab-simulated storage. Moreover, X-ray diffraction (XRD) test was conducted to reveal the interaction mechanism among crumb rubber, nanoclay and neat bitumen. It is expected that the overall findings of this study would be useful for selecting additives to provide enhanced storage stability of asphalt rubber.

## 2. Materials and Methods

### 2.1. Materials

Asphalt binder with a penetration grade of 60/70 (Pen60/70) commonly used in south China was applied as neat bitumen to prepare AR and nanoclay-CRM-modified asphalt (ARN). AR binder was prepared by high shear (10,000 rpm) blending 40-mesh CRM with neat bitumen at 180 °C for 1 h. Two different CRM dosages (10 wt. % and 20 wt. % by neat bitumen) were used, referring to low and high rubber dosages. For ARN binder preparation, certain percentages of CRM and nanoclay (3 wt. % by neat bitumen) were combined and then incorporated to hot asphalt binder by the same blending process of AR. In addition, to evaluate the individual effect of nanoclay, the nanoclay-modified bitumen was also prepared by the same mixing process. For each blending process, about 800 g modified binder was prepared.

**Figure 2:**
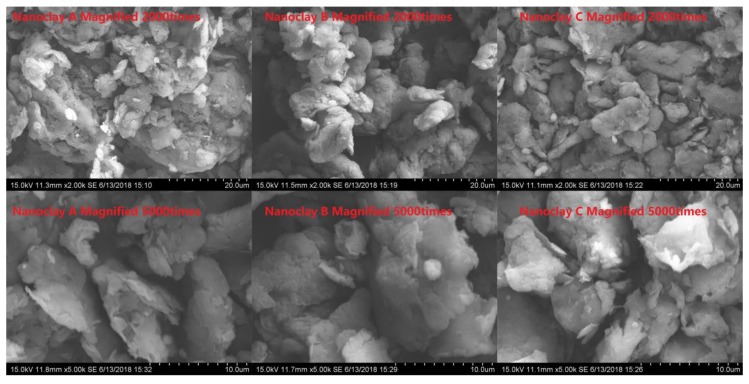
Nanoclay morphology

Three different types of nanoclay were used, which are labelled as A, B and C in this study. Figure 2 shows the morphologies of nanoclays in micro scale and Table 1 describes their physical properties. Among the three nanoclays, A is pure MMT with Na^+^ inorganic group, while B and C are MMTs containing inorganic groups exchanged with different alkyl ammonium ions. The detailed information on samples and the preparation process are summarized in Table 2.

### 2.2. Testing Program

The physical properties of test binders were characterized by penetration [23] and softening point [24] tests. The workability of AR and ARN samples were evaluated by rotational viscosity [25] using a Brookfield viscometer (RVDVII+). The rheological properties were evaluated by Superpave tests, including rutting factor (G^*^/sin δ) [26] test, multiple stress creep recovery (MSCR) tests [27], fatigue factor (G^*^sin δ) [26] test, linear amplitude sweep (LAS) test [28] and bending beam rheometer (BBR) [29] test.

The rutting resistance was characterized by two properties: the rutting factor (unaged samples) and the non-recoverable creep compliance (short-term aged samples). The short-term aging process was conducted by Rolling Thin Film Oven (RTFO). The rutting factor test started at 64 °C, and the testing temperature was raised automatically to the next PG temperature if the measured rutting factor passed the requirement of AASHTO M320. The non-recoverable creep compliance was determined by MSCR test using RTFO-aged samples. In each cycle of this test, a creep load was applied for 1 s followed by 9 s recovery at 64 °C. Each specimen was subjected to 10 cycles with a creep stress of 0.1 kPa, followed by 10 cycles with a creep stress of 3.2 kPa [27].

The fatigue resistance was characterized by both G^*^sin δ and LAS tests using RTFO-aged and Pressure Aging Vessel (PAV)-aged samples. G^*^sin δ test consisted of a temperature sweep starting at 25 °C with a decrement of 3 °C until the fatigue factor was larger than 5000 kPa. LAS test consisted of a frequency sweep ranging from 0.2 to 30 Hz at a strain level of 0.1%, followed by an amplitude sweep over a range of 0–30% strain at a frequency of 10 Hz [28].

The BBR test was conducted to evaluate the low-temperature performance of the binders according to AASHTO T313. Long term-aged (RTFO- and PAV-aged) samples were tested in a temperature fluid bath with a constant load (980 ± 50 mN) [29].

For storage stability characterization, a lab-simulated high-temperature storage process was conducted on test binders prior to the storage stability test [30]. About 70 g of hot asphalt binder was firstly poured into an aluminum tube (d = 25 mm, h = 140 mm). The tube was then vertically stored in an oven at 163 °C for 48 h. The tube was then cooled down in a refrigerator at 5 °C and cut into three equal parts horizontally. The top and bottom parts were used for rheological property and softening point analysis to identify their difference after storage.

The storage stability was characterized by two parameters. One was the traditional parameter—i.e., the difference in softening points [16,17]—and the other is separation index proposed by SHRP specification [31,32,33]. ASTM D5892 specifies that if the difference in the softening points between the top and the bottom sections is less than 2.5 °C, the sample is considered to have good high-temperature storage stability. However, this parameter has been questioned by some studies, as softening point is not a sensitive parameter that is able to detect the variation in binder components. Therefore, in addition to the difference in softening point, the SHRP specification defines the following separation index (SI) based on the complex shear modulus measurement:

(2) $$SI=\left(\frac{Max(G_{Top}^{*},G_{Bottom}^{*})-G_{Avg}^{*}}{G_{Avg}^{*}}\right)\times 100\;$$

where $G_{Top}^{*}$ and $G_{Bottom}^{*}$ are the complex shear moduli of the bottom and top parts after storage, respectively, at the evaluated temperature at a frequency of 10 rad/s, and $G_{Avg}^{*}$ is the average of $G_{Top}^{*}$ and $G_{Bottom}^{*}$.

Finally, the X-ray diffraction (XRD) test was conducted to monitor the micro-structure variation of nanoclay after it was mixed with neat bitumen and crumb rubber. Table 3 shows the experimental framework and the information of conducted tests, respectively.

## 3. Results and Discussion

### 3.1. Physical Properties

**Figure 3:**
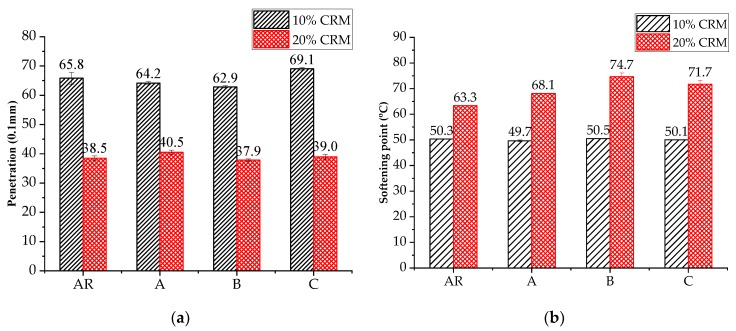
Physical properties of asphalt binders: (**a**) penetration; (**b**) softening point.

Figure 3 shows the penetration and softening point results of test binders. It is noted that AR20 had lower penetration and a higher softening point compared to AR10, indicating high CRM content results in higher stiffness and superior performance at high temperature. A previous study showed that nanoclay had an obvious impact on the penetration value of neat bitumen [34], however, for rubberized binders, the effect was not very significant. One possible reason is that the effect of CRM played a dominant role in modified binder stiffness, making the influence of nanoclay less noticeable. It is also interesting to note that nanoclay increased the softening point of AR20, but had no obvious effect on that of AR10.

### 3.2. Workability

**Figure 4:**
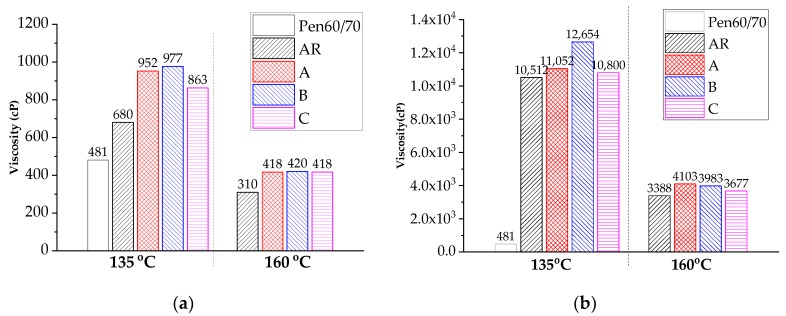
Rotational viscosity of asphalt binders: (**a**) Bitumen with 10 wt. % CRM; (**b**) Bitumen with 20 wt. % CRM.

The workability of modified bitumen was characterized based on rotational viscosity values—lower viscosity indicates better workability. The selected test temperatures of all asphalt samples were 135 °C and 160 °C. Figure 4a,b describe the viscosity values of the modified bitumen samples blended with 10 wt. % and 20 wt. % CRM, respectively. As expected, the incorporation of CRM made the binder more viscous. The incorporation of nanoclay further increased the viscosity values of AR10, but had limited influence on the workability of AR20 binder. According to Figure 4a, it is difficult to blend the AR20 binders (with and without nanoclay) with aggregate at even 160 °C. Warm mix asphalt (WMA) technology is recommended for those binders. More advanced nanoclays containing surfactants may also alleviate the workability and storage stability concerns of AR. Future study is recommended on developing such types of new nanoclay material.

### 3.3. Rutting Resistance

#### 3.3.1. Rutting Factor

**Figure 5:**
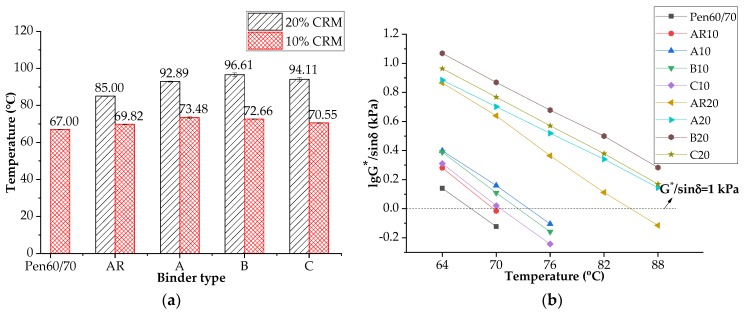
Rutting performance of asphalt binders: (**a**) failure temperatures; (**b**) Logarithm of G^*^/sin δ values (rutting factors).

A Superpave rutting factor (G^*^/sin δ) test was conducted for rutting resistance evaluation. The starting temperature was set at 64 °C. The testing temperature automatically rose 6 °C when the G^*^/sin δ exceeded the threshold value, which is 1.0 kPa for unaged binder. Figure 5a shows the failure temperatures of test binders and Figure 5b presents the G^*^/sin δ values’ variation with temperature. It is noted that the critical temperature of AR10 and AR20 were about 3 °C and 18 °C higher than that of the neat bitumen, respectively, showing improvement in high temperature performance. Since 3 °C increment in failure temperature is not an attractive modification effect, higher CRM dosage is more recommended for rutting. The incorporation of nanoclay further improved the rutting resistance. Nanoclay B had the most significant effect with AR20 while nanoclay A worked best with AR10. One possible explanation is that when nanoclay and CRM were used together, the nanoclay effect was reduced in comparison with that of crumb rubber. With the aid of nanoclay, the AR10 binders barely met the requirement of PG70.

#### 3.3.2. Creep Compliance

Table 4 shows the test results in terms of J_nr_, % recovery, and traffic levels determined according to AASHTO TP70-13. It is noted that all modified binders blended with 20 wt. % CRM exceeded the maximum allowable J_nr_ difference, i.e., 75%. This is mainly due to the extremely low J_nr_ values at 0.1 kPa. However, the low J_nr_ at 0.1 kPa and 3.2 kPa still ensured adequate resistance to permanent deformation at high temperature. By comparison, the J_nr_ values of AR10 and AR20 were about 1/2 and 1/47 of that of neat bitumen, respectively. Adding nanoclay further improved the rutting resistance of AR.

### 3.4. Fatigue Resistance

#### 3.4.1. Fatigue Factor

**Figure 6:**
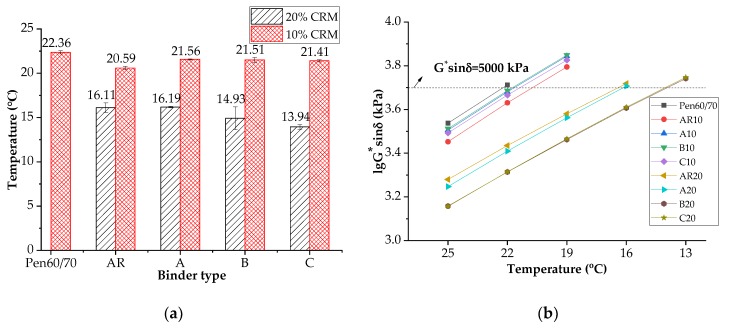
Fatigue performance of asphalt binders: (**a**) failure temperatures; (**b**) Logarithm of G^*^sin δ values (fatigue factors).

The fatigue resistance of asphalt binders was evaluated by Superpave fatigue factor (G^*^sin δ) through a temperature sweep starting at 25 °C with a decrement of 3 °C until the fatigue factor exceeded 5000 kPa. Lower G^*^sin δ values indicate superior fatigue resistance. Figure 6a,b show the results of fatigue failure temperature and the G^*^sin δ variation with decreasing temperatures. It is noted that the failure temperature of AR10 was approximately 1.8 °C lower than that of Pen60/70 but 4.4 °C higher than AR20. More CRM dosages led to both superior rutting and fatigue resistance. According to Figure 6, the nanoclays caused a slight deterioration of the fatigue resistance of AR10, but nanoclays B and C showed certain positive effects on the fatigue resistance of AR20. In particular, nanoclay C enhanced the fatigue resistance most effectively by decreasing the failure temperature by 2.2 °C.

#### 3.4.2. LAS Fatigue Life

**Figure 7:**
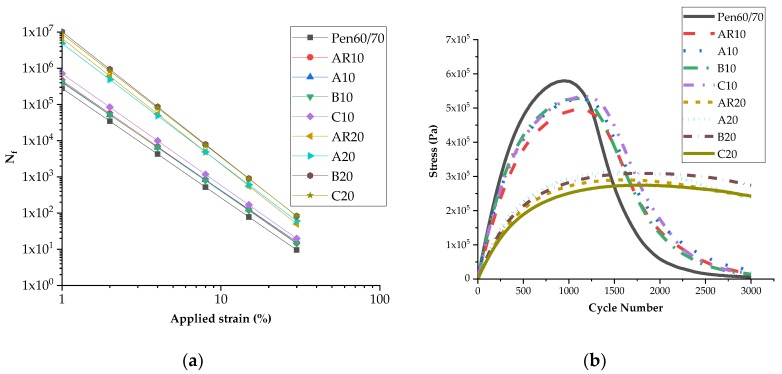
LAS test results: (**a**) fatigue life curves; (**b**) stress amplitude curves.

A previous study [35] showed that the LAS test is more practical than the existing performance criteria of G^*^sin δ. In this study, the LAS test facilitated the evaluation of the complex behavior of the binder at a wide range of loading levels. Table 5 presents the value of A and B obtained from the LAS test for the binders considered in this study at 25 °C—A indicates the fatigue life of the binder at 1% strain amplitude, whereas B is a measurement of strain susceptibility of the binders, i.e., the rate of reduction in fatigue life with an increase in strain. It was observed that the fatigue life of AR10 and AR20 was approximately 1.6 times and 24 times higher than that of neat bitumen, respectively, and nanoclays B and C showed certain positive effects on the fatigue life of AR20, which was consistent with the results of the fatigue factor test. However, this is different from nanoclay C, which increased the fatigue resistance of AR10. It can also be seen that the B value of AR10 is close to that of neat bitumen and 0.45 times higher than that of AR20, indicating a higher strain susceptibility of AR20. Additionally, the fatigue lives at different applied strains were calculated to indicate the N_f_ variation as shown in Table 5 and Figure 7a. It was noticed that AR20 had a higher fatigue life than neat bitumen and AR10 at a wide range of loading levels, from 1% to 30%. The stress amplitude curves also indicated a higher fatigue life of AR20 than AR10 and neat bitumen, as shown in Figure 7b, for lower stress at the same and higher cycle numbers at the peak of stress curve. Therefore, the effect of strain susceptibility could be compensated by a sufficiently high fatigue life. Although LAS and G^*^sin δ test results were not completely consistent with each other, the fatigue test results proved that the negative effect of selected nanoclays on fatigue resistance was negligible.

### 3.5. Low Temperature Cracking Resistance

Table 6 shows the results of BBR test for all binders. According to the AASHTO specification, the stiffness value should be less than ≤ 300 MPa, while the m-value should be larger than ≥ 0.3 for a specific temperature grade. As the BBR test results indicate, all modified binders met the requirements at −12 °C. It can also be noted that adding CRM decreased the creep stiffness of neat bitumen and adding nanoclay further decreased the stiffness value of AR modified binder. Therefore, it is believed that the nanoclay-AR binder can be applied in colder regions since both the CRM and nanoclay provide superior resistance to low-temperature cracking.

### 3.6. Storage Stability

#### 3.6.1. Effects of Nanoclay on Neat Bitumen

**Figure 8:**
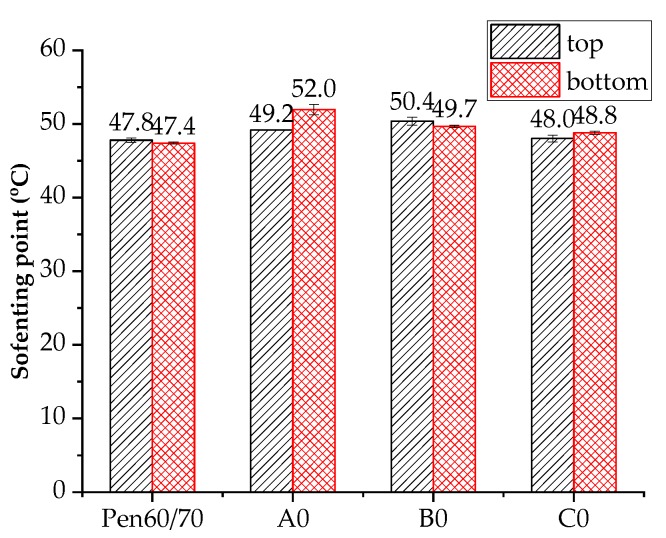
Storage stability of nanoclay modified bitumen.

Figure 8 shows the storage performance of nanoclay-modified bitumen (A0, B0 and C0) evaluated by softening point difference. The smaller the difference in softening point value (D-value), the better storage stability the modified bitumen has. Although the nanoclays have higher specific gravity (1.7–1.8) than neat bitumen (1.06), the storage stability of nanoclay modified binders was acceptable. In particular, for B0 and C0 binders, the softening point difference was ≤0.8 °C. These results indicate a relatively stable dispersion of nanoclay in liquid bitumen. The movement of solid particles (nanoclay) in the liquid bitumen depends on the difference in density, but also on the dimension and surface area of nanoclay particles. The stable dispersion is attributed to two aspects, i.e., the colloidal size and the intercalated layer structure of nanoclay. Driven by van der Waals forces, the nano materials move randomly in the bitumen fractions rather than directly ascend (or descend) in vertical direction [36]. Furthermore, the bitumen fractions penetrate into the nanoclay layers, modifying the original structure to intercalated or exfoliated structure within bitumen components, making the nanoclay particles stably disperse in hot liquid bitumen.

#### 3.6.2. Effects of Nanoclay on AR

**Figure 9:**
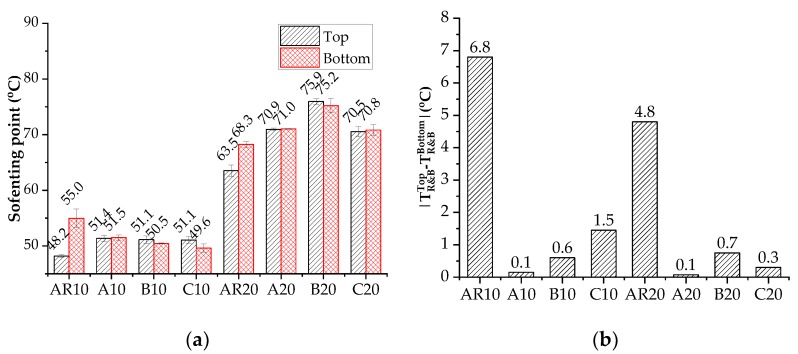
Softening point difference of ARs and ARNs after storage: (**a**) Softening points of top and bottom sections; (**b**) D-value between top and bottom sections.

Figure 9 presents the softening point difference of rubberized binders after lab-simulated storing process. As expected, both AR10 and AR20 binder had poor storage stability. The softening point difference value (D-value) between the top and bottom parts of asphalt binder was 6.8 °C and 4.8 °C for AR10 and AR20, respectively. The incorporation of nanoclay decreased such difference to acceptable values (<2.5 °C). Among the three nanoclays, nanoclay A seemed to show the best storage stability enhancing effect as the D-value was only 0.1 °C for both the low and high CRM dosage cases.

In addition to the traditional evaluating method, a new characterizing parameter, called separation index (SI, Equation (2)), as proposed by Abdelrahman et al. [31] was used in this study.

Table 7 and Table 8 present the SI values at different testing temperatures. To alleviate potential errors (same performance but different material components), the SI value was tested at three temperatures, including: a typical intermediate temperature (25 °C), a typical high temperature (64 °C), and the rutting failure temperature (70 °C for AR10 group and 82 °C for AR20 group). As shown in Table 7 and Table 8, it is noticed that the SI values of AR10 at high temperature and the SI values of AR20 at low temperature were much greater than the SI values of other specimens. The incorporation of nanoclay reduced the SI value of both AR10 and AR20. Being consistent with the D-value findings, nanoclay A showed the best performance in enhancing storage stability.

The temperature sensitivity parameter, *k* in Equation (3), was also determined from the complex shear modulus test results. In Equation (3), *T* represents the test temperature and *b* is a constant. A best-fit straight line was applied to a plot with logG^*^ on the vertical axis and *T* on the horizontal axis to determine *k* (slope of the best-fit straight line).

(3) $$\log G^{*}=kT+b\;$$

The separation index of *k* was calculated based on Equation (2); the computed results are shown in Table 9. It is evident from Table 9 that the SI(*k*) values of all ARNs are far smaller than the SI(*k*) values of ARs, which further indicated that the storage stability of AR was improved in the presence of nanoclay.

Both of the softening point and complex modulus evaluation showed that the selected nanoclays have an obvious effect on enhancing the storage stability of AR. According to the softening point evaluation, nanoclay A worked best with both AR10 and AR20 binders. By comparison, the complex modulus results indicated that nanoclay A showed optimum effect on AR20 binder, while nanoclay B worked best with AR10 binder. Moreover, the satisfied improved performance of nanoclay A was consistent with the complex modulus evaluation results at different temperatures.

### 3.7. Mechanism Investigation

**Figure 10:**
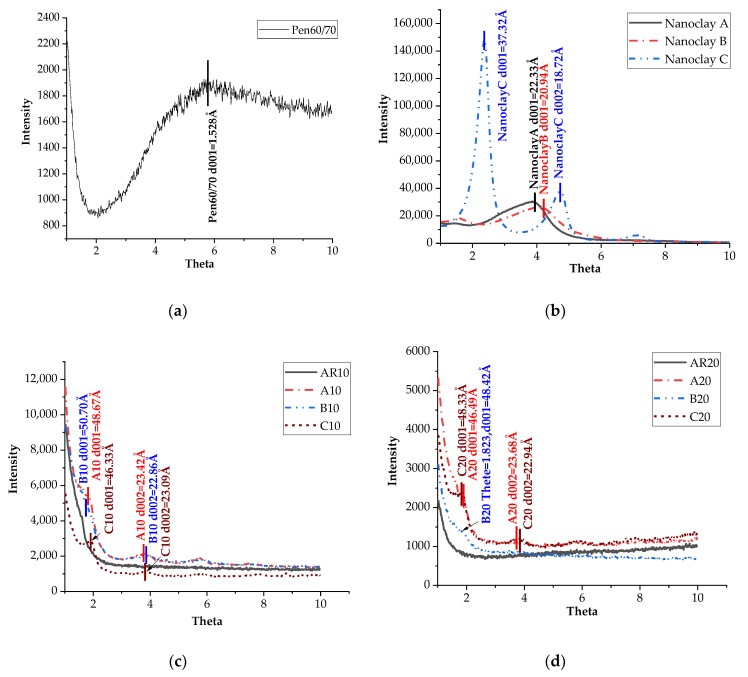
XRD evaluation: (**a**) neat bitumen; (**b**) nanoclay A, B and C; (**c**) ARNs with 10 wt. % CRM; (**d**) ARNs with 20 wt. % CRM

To investigate the working mechanism of each type of nanoclay when mixed with CRM and asphalt binder, XRD test was conducted to monitor the distance between the layers of nanoclays. The results of XRD test are shown in Figure 10. Based on the XRD analysis, the basal interlayer spacing (*d*) can be calculated from the first strong peak in the XRD spectra by means of the following equation:

(4) 2dsinθ=λ 

where *θ* is the diffraction angle and *λ* is the wavelength of the diffractometer (0.154 nm).

The interlayer spacing of neat bitumen sample was also calculated based on Equation (4) and is presented in Figure 10a. It was observed that the d001 of neat bitumen is quite small at the selected scope of angle. In addition, Figure 10b shows that nanoclays A and B have only one peak while nanoclay C has two peaks at different positions. Nanoclay C had the largest gap distance between the platelets while B had the smallest. According to Galooyak et al. (18), nanoclay, after being mixed with bitumen, can act like regular particulate fillers if the distances between the clay platelets remain the same. In that case, the polymer cannot enter the layer structure of nanoclays. In contrast, if the layer distances are increased, it indicates that the polymer chains have penetrated into the nanolayers, and the intercalated structures get established.

Figure 10c,d show the XRD spectra of the test binders modified with both CRM and nanoclays. Peaks can be noticed in the curve of all modified binders prepared with nanoclay except B20, while no peak was found for the binders with CRM only. One possible reason is that the signal of nanoclay B was hindered by the diffraction caused by the increasing CRM, as the XRD spectra peak of nanoclay B is relatively low. A tiny peak can also be observed around the position where theta equals 1.823; thus, d001 of B20 can be thought as 4.842 nm. Table 10 summarizes the XRD results. It was noticed that the clay interlayer diffraction peak in ARNs shifted toward lower angles compared to the original nanoclays, which is consistent with a previous study [31].

The modification mechanism of nanoclays on storage stability can be explained as follows. Neat bitumen is usually regarded as a dynamic colloidal system consisting of a suspension of high molecular weight asphaltene micelles dispersed in a lower molecular weight oily medium (maltenes) [37]. Crumb rubber particles absorb maltenes in neat bitumen and swell, but cannot be totally dissolved. The density difference between crumb rubber and components in neat bitumen leads to precipitation of rubber particles [38,39]. With nano sizes and layer structures, the nanoclays do not settle down easily in the neat bitumen even though their densities are larger than that of neat bitumen. The existence of nano layers provides resistance to prevent the rubber particles from precipitating, resulting in more uniform distribution of crumb rubber in neat bitumen, thus enhancing the storage stability of AR binder.

## 4. Conclusions

This study evaluated the feasibly of using nanoclay to alleviate the storage stability problem of AR binders. Three types of nanoclays were blended with AR with low and high rubber dosages (10% and 20%). The rheological properties and storage stability of rubber-nanoclay-modified binder samples were tested and compared. Based on laboratory test results, the following findings were obtained:

- Adding nanoclay slightly increased the viscosity of AR, but had insignificant effects on its rutting, fatigue and low-temperature properties.
- The addition of all three types of nanoclays to AR had obviously positive effects on the storage stability of AR binder, for both high and low CRM dosages.
- Nanoclay A, which is pure montmorillonite with Na^+^ inorganic group, exhibited the most significant effect in enhancing storage stability.

The findings of this study have revealed that nanoclay can be incorporated in AR binder to enhance its storage stability. Further studies will be conducted to more comprehensively evaluate the interaction mechanism among nanoclay, crumb rubber and neat bitumen from a microscopic perspective. Moreover, the industrial scale evaluation for longer storage periods is recommended for future research.

## Figures and Tables

**Table 1:** General information of selected nanoclay additives.

Nanoclay ID	A	B	C
**Composition**	Pure MMT with Na^+^ inorganic group	Pure MMT with Hydroxyl organic ammonium	Pure MMT with Double alkyl ammonium
**Specific gravity**	1.8	1.8	1.7
**Bulk gravity**	≤0.3	≤0.3	≤0.3
**Hydrophilicity**	Medium	Strong	Poor
**X-ray d001**	2.24 nm	2.09 nm	3.73 nm
**Applicable polymer**	PE, PP, PVC	N/A	PP and other thermos plasticity polymers

**Table 2:** Composition of different modified asphalt rubber samples

Binder Type	Modifier	Dosage of Crumb Rubber (wt. %)	Dosage of Nanoclay (wt. %)
Pen60/70	N/A	N/A	N/A
AR10	CRM	10	N/A
A10	CRM, Nanoclay A	10	3
B10	CRM, Nanoclay B	10	3
C10	CRM, Nanoclay C	10	3
AR20	CRM	20	N/A
A20	CRM, Nanoclay A	20	3
B20	CRM, Nanoclay B	20	3
C20	CRM, Nanoclay C	20	3
A0	Nanoclay A	N/A	3
B0	Nanoclay B	N/A	3
C0	Nanoclay C	N/A	3

**Table 3:** Details of laboratory tests.

Performance Property	Tests	Aging Level	Specification/Standard	Notes
**General property**	Penetration	unaged	ASTM D5	N/A
	Softening point		ASTM D36	N/A
**Workability**	Rotational viscosity	unaged	AASHTO T316	135 °C & 160 °C
**Rutting resistance**	Rutting factor (G*/sin δ)	unaged	AASHTO M320	2 mm gap; 25 mm plate; beginning at 64 °C
	MSCR	RTFO aged	AASHTO MP19-10	2 mm gap; 25 mm plate; 64 °C
**Fatigue resistance**	Fatigue factor (G*sin δ)	RTFO + PAV aged	AASHTO M320	2 mm gap; 8 mm plate; beginning at 25 °C
	LAS		AASHTO TP101	2 mm gap; 8 mm plate; 25 °C
**Storage stability**	Softening point	unaged	ASTM D36	N/A
	Complex shear modulus		AASHTO M320	25 mm plate; 25 °C, 64 °C, 70 °C and 82 °C
**Low temperature cracking resistance**	BBR	RTFO + PAV aged	AASHTO T313	−12 °C, −18 °C, −24 °C
**Internal layer distance of nanoclay**	XRD	unaged	N/A	N/A

**Table 4:** MSCR test results.

Binder Type	J_nr_			% Recovery	
	@ 0.1 kPa (kPa^−1^)	@ 3.2 kPa (kPa^−1^)	J_nr_% Diff	@ 0.1 kPa (kPa^−1^)	@ 3.2 kPa (kPa^−1^)
**Pen60/70**	3.988	4.586	15.0	2.1	−0.5
**AR10**	1.723	2.230	29.4	10.4	2.1
**A10**	1.635	2.145	31.2	11.4	1.9
**B10**	1.548	2.105	36.0	13.1	1.9
**C10**	1.470	2.117	44.0	15.6	1.9
**AR20**	0.019	0.098	402.6	91.6	63.0
**A20**	0.034	0.168	392.7	89.0	55.8
**B20**	0.013	0.090	608.0	94.6	67.1
**C20**	0.023	0.141	518.4	92.0	59.5

**Table 5:** LAS test results.

Binder Type	A	B	Applied Strain (%)				
			2	4	8	15	30
**Pen60/70**	2.81 × 10^5^	−3.02	3.46 × 10^4^	4.26 × 10^3^	5.24 × 10^2^	7.83 × 10^1^	9.64 × 10^0^
**AR10**	4.63 × 10^5^	−3.04	5.63 × 10^4^	6.86 × 10^3^	8.34 × 10^2^	1.23 × 10^2^	1.50 × 10^1^
**A10**	4.07 × 10^5^	−2.98	5.16 × 10^4^	6.56 × 10^3^	8.33 × 10^2^	1.28 × 10^2^	1.63 × 10^1^
**B10**	4.30 × 10^5^	−3.03	5.27 × 10^4^	6.46 × 10^3^	7.92 × 10^2^	1.18 × 10^2^	1.45 × 10^1^
**C10**	7.17 × 10^5^	−3.09	8.45 × 10^4^	9.95 × 10^3^	1.17 × 10^3^	1.69 × 10^2^	1.99 × 10^1^
**AR20**	6.74 × 10^6^	−3.47	6.08 × 10^5^	5.48 × 10^4^	4.93 × 10^3^	5.56 × 10^2^	5.01 × 10^1^
**A20**	4.88 × 10^6^	−3.32	4.89 × 10^5^	4.89 × 10^4^	4.89 × 10^3^	6.07 × 10^2^	6.08 × 10^1^
**B20**	1.02 × 10^7^	−3.45	9.39 × 10^5^	8.61 × 10^4^	7.90 × 10^3^	9.04 × 10^2^	8.29 × 10^1^
**C20**	8.87 × 10^6^	−3.40	8.40 × 10^5^	7.96 × 10^4^	7.54 × 10^3^	8.89 × 10^2^	8.43 × 10^1^

**Table 6:** BBR test results.

Binder Type	−12 °C		−18 °C	
	Stifness (MPa)	m-Value	Stifness (MPa)	m-Value
**Pen60/70**	280	0.280	541	0.199
**AR10**	274	0.301	527	0.208
**A10**	208	0.307	437	0.217
**B10**	241	0.301	482	0.207
**C10**	224	0.300	432	0.204
**AR20**	166	0.347	321	0.239
**A20**	110	0.338	220	0.268
**B20**	89.1	0.358	209	0.299
**C20**	105	0.348	238	0.268

**Table 7:** Complex shear modulus and separation index of different asphalt binders at low and high temperatures.

Binder Type	25 °C			64 °C		
	Top (kPa)	Bottom (kPa)	SI	Top (kPa)	Bottom (kPa)	SI
**AR10**	893.184	915.579	1.24	1.952	4.856	42.66
**A10**	874.587	836.958	2.20	2.884	2.874	0.17
**B10**	873.405	889.83	0.93	2.781	2.777	0.07
**C10**	812.966	755.015	3.70	2.725	2.343	7.54
**AR20**	1458.138	718.015	34.01	10.888	10.838	0.23
**A20**	1065.133	1008.977	2.71	10.69	10.71	0.09
**B20**	1062.84	961.9	4.99	14.545	15.166	2.09
**C20**	886.238	970.832	4.56	11.611	12.159	2.31

**Table 8:** Complex shear modulus and separation index of different asphalt binders at high critical temperature of AR.

Binder Type	70 °C			Binder Type	82 °C		
	Top (kPa)	Bottom (kPa)	SI		Top (kPa)	Bottom (kPa)	SI
**AR10**	0.927	2.422	44.64	AR20	2.576	2.715	2.63
**A10**	1.377a	1.416	1.40	A20	2.905	2.895	0.17
**B10**	1.364	1.379	0.55	B20	4.143	4.242	1.18
**C10**	1.344	1.136	8.39	C20	3.102	3.37	4.14

**Table 9:** Temperature sensitivity evaluation results for different asphalt binders.

Binder Type	*k* (Top)	*k* (Bottom)	SI (*k*)
**AR10**	−0.0670	−0.0577	7.4868
**A10**	−0.0628	−0.0622	0.4856
**B10**	−0.0630	−0.0631	0.1014
**C10**	−0.0624	−0.0633	0.7087
**AR20**	−0.0493	−0.0432	6.6247
**A20**	−0.0460	−0.0456	0.4637
**B20**	−0.0432	−0.0421	1.2228
**C20**	−0.0439	−0.0441	0.1520

**Table 10:** Layer distance of nanoclay before and after mixed with bitumen.

Nanoclay Type	Original Layer Distance (Å)	Measured Layer Distance in AR10 (Increment) (Å)	Measured Layer Distance in AR20 (Increment) (Å)
**Nanoclay A**	22.33	48.46 (26.13)	46.49 (24.16)
**Nanoclay B**	20.09	50.70 (30.61)	48.42 (28.33)
**Nanoclay C**	37.32	46.33 (9.01)	48.33 (11.01)
